# *IL-10* A-Allele as a Biomarker for Periodontitis Severity in Bulgarian Patients

**DOI:** 10.3390/genes15091221

**Published:** 2024-09-18

**Authors:** Zdravka Pashova-Tasseva, Velitchka Dosseva-Panova, Antoaneta Mlachkova, Alexey Savov, Ekaterina Tosheva

**Affiliations:** 1 Department of Periodontology, Faculty of Dental Medicine, Medical University of Sofia, 1000 Sofia, Bulgaria; v.doseva@fdm.mu-sofia.bg (V.D.-P.); a.mlachkova@fdm.mu-sofia.bg (A.M.); 2 National Genetic Laboratory, University Hospital of Obstetrics, Medical University, 1000 Sofia, Bulgaria; asavov@medfac.mu-sofia.bg; 3 Department of Statistics and Econometrics, Faculty of Applied Informatics and Statistics, University of National and World Economy, 1000 Sofia, Bulgaria; etosheva@unwe.bg

**Keywords:** periodontitis, single-nucleotide polymorphism, Interleukin 10, biomarker, risk factor, genetic susceptibility, RFLP analysis, epidemiology of periodontitis

## Abstract

Background: Periodontitis is a complex disease, and bacterial factors play a crucial role in its initiation. The contributions of genetic and epigenetic factors to the pathogenesis of periodontal disease are increasingly recognized. Single-nucleotide polymorphisms (SNPs) in various molecules, including cytokines, are of particular interest due to their established involvement in numerous diseases. This study investigates the influence of SNPs in the *IL-10* gene at positions *−592 (rs1800872) C>A* and *−1082 (rs1800896) T>C* (also referred to as *1082A>G*) on the severity of periodontitis in a cohort of Bulgarian patients. Methods: In the recent study, both clinical and paraclinical methodologies were employed to comprehensively assess the periodontal status of the participants. The genotypic characterization of *IL-10* polymorphisms was performed by PCR RFLP analysis. Statistical analyses, including principal component analysis (PCA), were executed utilizing IBM SPSS Statistics Version 21. Results: We have established a statistically significant association between the presence of at least one A-allele in the patients’ genotype and the incidence of severe periodontitis (*p* = 0.047). Conclusions: *IL-10* single-nucleotide polymorphisms (SNPs) could be effectively considered as biomarkers for the severity of periodontitis.

## 1. Introduction

Periodontitis affects populations globally, with its incidence increasing with advancing age [1]. The aging population, and the consequent expansion in the number of individuals who are edentulous due to periodontitis, represents a significant socio-economic challenge. Periodontitis is the sixth most prevalent condition globally, with its severe forms (Stage III and IV) affecting approximately 10% of the adult population [2,3,4,5]. Advanced periodontal disease is the primary etiology of adult tooth loss, often requiring extensive dental interventions such as extractions, dental implants, or prosthetic rehabilitation. These procedures can be both financially burdensome and time-consuming for patients and dental practitioners alike [6]. Periodontal infection is initiated by pathogenic bacterial species that trigger an inflammatory response. The presence of the periodontal pathogens stimulates the hosts immune response which results in the destruction of the essential components of the periodontal apparatus—alveolar bone and periodontal ligament [7]. The progression to periodontal disease occurs when the host’s immune response is exacerbated by anaerobic Gram-negative bacteria within the bacterial plaque biofilm [8]. While pathogenic microorganisms are regarded as the primary etiological factors in periodontitis, additional risk factors such as smoking and diabetes also play a significant role in disease development [9].

The significance of the genetic factor in relation to periodontitis is not sufficiently clear. Genetic and immunological differences between individuals may be important risk factors for periodontitis [10]. Genetic factors can substantially influence host susceptibility to periodontitis. These genetic factors also play a role in the pathogenesis of various complex diseases, including periodontitis. 

Numerous molecules play critical roles in the onset and progression of chronic diseases, such as periodontitis. Among these, cytokines—both pro-inflammatory and anti-inflammatory—have been extensively studied due to their fundamental role in the host immune response. In addition to cytokines, several other molecules contribute to these processes, including tumor necrosis factor-α (TNF-α), matrix metalloproteinases (MMPs), and various immune and epithelial cells. Key cytokines such as IL-1, IL-2, IL-6, IL-10, IL-13, and IL-17, among others, significantly influence the host’s response to bacterial challenges [11,12,13]. The pathogenic bacterial species directly damage the periodontal tissues. Additionally, these bacteria produce lipopolysaccharides that stimulate the production of inflammatory mediators, including cytokines, which in turn activate immune cells. These processes disrupt the host’s immune response, leading to the progression of periodontal disease, which is characterized by tissue destruction, including damage to the periodontal ligament and alveolar bone [14,15]. 

Genetic factors significantly influence periodontitis, particularly those related to host susceptibility such as cytokine genes, cell surface receptors, chemokines, enzymes, and others. Research indicates that polymorphisms in interleukins can affect the development of periodontitis, with genetic variations potentially having both detrimental and protective impacts. Variations in immune cell development and antigen presentation may contribute to an individual’s risk of developing autoimmune or inflammatory diseases. Cytokine gene polymorphisms play a crucial role in determining the clinical expression and progression of periodontal disease, with single-nucleotide polymorphisms (SNPs) including cytokines serving as valuable tools for identifying risk alleles at the population level. Cytokines are vital for maintaining tissue homeostasis by regulating immune cell recruitment, pathogen activity, and osteoclast function, which in turn affects the intensity and duration of the immune response. Proinflammatory cytokines enhance bacterial phagocytosis, attract immune cells to sites of inflammation, promote the maturation of dendritic cells, and guide the immune response to bacterial invasion. In contrast, anti-inflammatory cytokines help modulate the inflammatory response and mitigate inflammation. The progression of periodontal disease begins with the stimulation of the innate immune response by periodontopathogens. These processes involve macrophages, NK cells, dendritic cells, neutrophils, and monocytes, which collectively produce proinflammatory cytokines. The adaptive immune response, driven by T- and B-lymphocytes, further exacerbates the condition by releasing proinflammatory molecules such as tumor necrosis factor-α (TNF-α), interferon-γ (IFN-γ), and cytokines like IL-1, IL-6, and IL-17. The so-called naïve CD4+ T cells differentiate into various subsets, including Th1, Th2, Th17, Treg, and Tfh cells, under different inflammatory conditions. Th1 cells, producing IFN-γ and IL-12, and Treg cells, producing TGF-β, IL-2, and IL-10, are associated with anti-inflammatory effects. Th2 cells, which secrete IL-4, IL-5, and IL-13, contribute to B-cell-mediated destruction in periodontitis. Th17 cells, producing IL-17 and IL-23, are known for their role in inflammation across several immune-mediated diseases such as psoriasis, rheumatoid arthritis, asthma, multiple sclerosis, inflammatory bowel disease, Alzheimer’s disease, etc. The chronic nature of periodontal disease arises from an imbalance between periodontopathogens and proinflammatory mediators, involving both innate and adaptive immune responses. This imbalance is maintained by a network of cytokines with opposing effects—proinflammatory cytokines like IL-1α, IL-6, IL-17, and TNF-α drive tissue damage, while anti-inflammatory cytokines such as IL-10 and IL-13 work to counteract these effects [16]. 

Interleukin-10 (IL-10) is a potent anti-inflammatory cytokine that modulates immune responses by stimulating T-cells and suppressing the activity of certain pro-inflammatory cytokines [9]. In the promoter region of *IL-10*, three polymorphisms including *rs1800871 (−819 T/C)*, *rs1800872 (−592 A/C),* and *rs1800896 (−1082 A/G)* are evaluated. Single-nucleotide polymorphisms (SNPs) in *IL-10* are of particular interest due to their association with various diseases characterized by dysbiosis. This is supported by research investigating the correlation between *IL-10* polymorphisms and periodontal disease in patients with severe periodontitis compared to healthy individuals/control group. These studies highlight the potential role of *IL-10* genetic variations in influencing the susceptibility to periodontal disease [16]. This statement is supported by studies comparing patients with severe periodontitis to healthy controls, which examine the association between *IL-10* polymorphisms and periodontal disease. Many of these studies have identified specific genotypes or haplotypes that are linked to increased susceptibility to severe periodontitis [17]. The role of IL-10 in mediating anti-inflammatory responses and suppressing periodontal pathogens has been extensively studied. This importance was highlighted in experiments using *IL-10*-deficient mice, which demonstrated a marked susceptibility to periodontitis induced by *Porphyromonas gingivalis* and exhibited pronounced pro-inflammatory phenotypes [18]. 

According to the existing literature, IL-10 is significantly associated with the risk of developing periodontitis. This association is attributed to IL-10’s role in modulating the immune response and influencing the inflammatory pathways involved in periodontal disease. As an anti-inflammatory cytokine, IL-10 helps regulate immune responses by inhibiting the production of pro-inflammatory cytokines and promoting a balanced immune environment. IL-10 has the capacity to inhibit the production of key inflammatory mediators, including matrix metalloproteinases (MMPs), the receptor activator of nuclear factor-kappa B (RANK), and its ligand, namely the receptor activator of nuclear factor-kappa B ligand (RANKL) [19,20]. IL-10 deficiency is linked to increased alveolar bone resorption and reduced bone formation. Single-nucleotide polymorphisms (SNPs) in *IL-10* can diminish the production of anti-inflammatory proteins. Consequently, low IL-10 levels lead to the inadequate suppression of pro-inflammatory cytokines and collagenases, adversely affecting bone structure in conditions such as osteoporosis and periodontitis. This impact is particularly pronounced in women, who are more susceptible to osteoporosis and experience further reductions in bone density. IL-10 is thus recognized as a crucial regulator of bone homeostasis [16]. 

Variations in the *IL-10* gene may affect an individual’s susceptibility to periodontitis by altering the efficacy of these regulatory mechanisms [21]. 

The distribution of *IL-10* single-nucleotide polymorphisms across different geographic regions shows distinct variations in genotype frequencies among the studied populations. However, the influence of these polymorphisms on the severity of periodontitis remains a topic of scientific debate [22]. 

The objective of the recent study is to investigate the impact of *IL-10* single-nucleotide polymorphisms (SNPs) on the severity of periodontitis in a cohort of Bulgarian patients.

## 2. Materials and Methods

The genetic polymorphisms for *IL-10* SNPs were investigated in both patients with periodontitis and individuals with a diagnosis for periodontal health in order to prove or rule out the role of these polymorphisms in the periodontal disease or periodontal health in a cohort of Bulgarian patients. The participants in the research were recruited from the private practices of the dental specialists conducting the study after detailed anamnesis was taken and periodontal examination was performed. All participants included in the study were referred either for treatment of their periodontal disease or for prophylactics of the periodontal health. The chairside diagnosis provided in the dental office was an argument for the enrollment of the participants in the study. Patients with gingivitis or mild periodontitis and patients with localized periodontitis were excluded from the research.

A total of 102 participants were enrolled in this study. The inclusion criteria for the periodontitis group were systemically healthy adults with periodontitis (stages II to III) [23], the presence of periodontal pockets with probing depths ≥7 mm, clinical attachment loss ranging from 3–4 mm to ≥5 mm, radiographically confirmed bone loss, at least 20 teeth present, and no periodontal treatment received in the past year. To establish diagnosis periodontitis, at least two sites with clinical attachment loss (CAL) ≥ 2 mm in two interproximal sites or CAL ≥ 3 mm in oral sites at ≥2 non-adjacent teeth must be identified which cannot be attributed to non-periodontal causes. In addition to the staging, the following criteria were considered:

- Stage II—severity factors: CAL 3 to 4 mm; radiographic bone loss reaching the coronal third (15% to 33%) of the root; no tooth loss due to periodontitis; complexity factors: maximum probing depth ≤5 mm; mostly horizontal bone loss.
- Stage III—severity factors: the greatest CAL ≥5 mm; radiographic bone loss extending to the mid-third of root and beyond tooth loss due to periodontitis ≤4 teeth; complexity factors: in addition to Stage II complexity—maximum probing depth ≥6 mm; vertical bone loss ≥3 mm; furcation involvement Class II or III, and moderate ridge defect [23].

Regarding the distribution of the CAL, only generalized cases (CAL > 30%) were included in this study. The grade of periodontitis is established based on primary criteria and modifying factors [23]. 

Recent research has emphasized the importance of the presence of clinical signs of severe periodontal disease, one of which is the presence of deep periodontal pockets. The deepest periodontal pockets, PD > 7, often represent a clinical challenge. They are associated with poor tooth prognosis, which renders difficult the treatment of the tooth or requires special surgical treatment including surgical procedures. Even after non-surgical therapy, adequate pocket reduction cannot be achieved, and residual periodontal pockets may be present [24]. A systemic review revealed a probing pocket depth ≥6 mm as the threshold for the surgical treatment of periodontal pockets. Another study has demonstrated that the deep periodontal sites could be considered a risk for the worsening of the prognosis in the site related to many factors [25,26]. The patients with a presence of residual pockets ≥5 mm are considered to be at a high risk of disease progression and patients with residual pockets with a pocket depth equal or greater than 7 mm are at risk of tooth loss [27,28].

For the healthy control group, the inclusion criteria were full mouth bleeding on probing score (FMBS) < 10%, and PPDs ≤ 3 mm with no clinical attachment loss [29]. 

The exclusion criteria were as follows: a systemic disease known to be associated with or modulating the development and progression of periodontitis (such as diabetes, hepatitis, and immunodeficiency viruses); patients undergoing immunosuppressive therapy or taking anti-inflammatory medications; and individuals who are pregnant or breastfeeding.

The clinical research methods included hygiene index FMPS and gingival index FMBS; bleeding on probing (BOP) index; probing pocket depth (PPD)*; clinical attachment level in mm (CAL)*. FMPS (full-mouth plaque score) and FMBS (full-mouth bleeding score) were performed simultaneously with the circumferential movement of the periodontal probe around all teeth and the results were assessed dichotomously evaluated. The probing pocket depth (PPD) was assessed by the insertion of the periodontal probe to the bottom of the pocket while sensing mild resistance. The values were calculated with the distance from the gingival margin to the bottom of the pocket/to the tip of the probe. The clinical attachment loss (CAL) is measured with the distance between the cemento-enamel junction and the bottom of the pocket. Bleeding in probing (BoP), an index representing the activity of the periodontal pocket, is registered dichotomously for each periodontal site [30,31,32,33,34].

*Measurements in mm were taken at 6 points for each tooth (mediobuccal, buccal, distobuccal, mediolingual, lingual, distolingual), with a manual periodontal probe CP15 (Hu Friedy). The data are registered in a periodontal card.

X-ray examination methods:

These aimed to provide paraclinical confirmation of the diagnosis “periodontitis” or “periodontal health” in the selected patients and test subjects. The following radiographic techniques were applied:

(1) Orthopantomography—analysis for the presence of bone loss, pattern of bone loss, any deviations in the bones, teeth, periarticular abnormalities, etc.
(2) Intraoral retroalveolar radiography—for the precise calculation of the Bl/Age ratio [23].

In patients with periodontitis, bone loss will be measured, specifically the bone loss/age ratio—Bl/age.

### 2.1. Laboratory Research Methods

The investigation and determination of gene polymorphism for Interleukin-10 (IL-10) at positions *(−1087)* and *(−592)* were performed by PCR amplification followed by re-striction enzyme digestion [35]. 

The DNA from all of the participants was collected with the ‘buccal mucosa sample’. The RFLP PRC analysis was performed in National Genetic Laboratory by the utilization of a Nucleo Spin MACHEREY-NAGEL kit with columns. The protocol for the isolation of genomic DNA from buccal mucosa was as follows:

- Remove the brush and spin the sample at 12,000 rpm for 10 min. Remove the supernatant and resuspend the cells with part of the water;
- Pre-lysis—add 180 μL T1 buffer and 25 μL proteinase K;
- Vortex and incubate at 56 °C for 1–3 h;
- Lysis—add 200 μL B3 buffer and incubate at 70 °C for 10 min;
- Add 210 μL 96–100% ethanol and vortex;
- The sample is transferred to the column and spun at 11,000× *g* for 1 min;
- Add 500 μL BW wash buffer and spin at 11,000× *g* for 1 min;
- Add 600 μL W5 wash buffer and spin at 11,000× *g* for 1 min;
- Dry spin at 11,000× *g* for 1 min;
- Elution of the sample—the column is placed in the pre-labeled 1.5 mL Eppendorf tube. Add 80 μL of BE buffer. Incubate at room temperature for 1 min and spin at 11,000× *g* for 1 min;
- Pre-analytical processing—PCR (polymerase chain reaction) and sample evaluation on 2% agarose gel.
- Analysis—RFLP (restriction fragment length polymorphism)—a method for detecting variants (polymorphisms/genetic markers) in the DNA molecule by the restriction of the DNA fragment using restriction enzymes that recognize the specified region. This results in the different length fragments of the PCR product and can thus be analyzed. Analysis is performed after separating the samples on a 3% agarose gel.

### 2.2. PCR Amplification

The DNA fragment containing the position *−592* was amplified in a 25 µL reaction mixture containing 100 ng of template DNA, 0.5 M of each primer, 1.5 mM of MgCl_2_, 200 M each of dGTP, dATP, dTTP, and dCTP, 2.5 unit of Taq polymerase and Taq polymerase buffer. The primers used were as follows: for the amplification of *−592* fragment, sense primer 5′gtgttcctaggtcacagtga, and antisense primer 5′gtcatggtgagcactacctga 3′. PCR was performed under the following cycling parameters: denaturation at 94 °C for 5 min, followed by 35 cycles of denaturation at 94 °C for 30 s; annealing at 60 °C for 30 s; and extension at 72 °C for 1 min. This was followed by final extension at 72 °C for 7 min.

The fragment containing the position −1082 was amplified in 25 µL reaction mixture containing 100 ng of template DNA, 0.5 M of each primer, 1.5 mM of MgCl_2_, 200 M each of dGTP, dATP, dTTP, and dCTP, and 2.5 unit of Taq polymerase and Taq polymerase buffer. The primers used were as follows for −1082, sense primer 5′ctcgctgcaacccaactggc 3′, and antisense primer 5′tcttacgcaacccaactggc 3′. PCR was performed under the following cycling parameters: denaturation at 94 °C for 5 min, followed by 35 cycles of denaturation at 94 °C for 30 s; annealing at 62 °C for 30 s; and extension at 72 °C for 30 s. This was followed by final extension at 72 °C for 7 min.

### 2.3. Restriction Fragment Length Polymorphism (RFLP)

The two alleles of the polymorphic site at the position −592 were identified by incubating a 15 µL aliquot of the PCR product with the specific restriction enzyme, followed by electrophoresis on agarose gels The reaction was carried out in a water bath for 16 h at 37 °C. The restriction enzyme RsaI cut the fragment at the position −592 when allele A was present, giving rise to 176 and 236 bp fragments.

The two alleles of the polymorphic site at the position 1082 were determined using of Mnl I restrictase. The enzyme was cut when the allele G was present and generated 106 and 33 bp fragments. The conditions were the same as in the previous assay.

The results of this restriction fragment length polymorphism assay were confirmed by the Sanger sequencing of the promoter region of the *IL-10* gene in the samples with different genotypes.

### 2.4. DNA Electrophoresis and Genotype Determination

The digested product was mixed with 1 μL of bromophenol blue and xylene cyanide, and electrophoretically separated on 3% agarose gel containing ethidium bromide (45 min at 95 V). Gels were observed under UV illumination.

### 2.5. Statistical Methods

Data were implemented by the statistical package PCA—IBM SPSS Statistics Version 21. *p* < 0.05 was chosen as the level of significance at which the null hypothesis is rejected. The following methods were applied:

1. Descriptive analysis—the frequency distribution of the considered signs, broken down by research groups, is presented in tabular form.
2. Pearson correlation analysis—to study the relationship between individual indicators.
3. Variation analysis—calculating estimates of central tendency and dispersion.
4. Principal component analysis (PCA)—to group indicators and patients.
5. Student’s *t*-test—for testing hypotheses about a difference between two independent samples.
6. Non-parametric Shapiro–Wilk test—to check the type of distribution.
7. Non-parametric Mann–Whitney test—for testing hypotheses of difference between two independent samples.

* Due to the inability to isolate DNA from some samples, it was necessary to recruit additional patients. Consequently, the results for the two gene polymorphisms are reported as follows: 102 participants for *IL-10* *−592* and 89 participants for *IL-10* −1082. 

All participants have signed an informed consent form approved by KENIMUS—Medical University Sofia, Bulgaria, with the following number and date of the ethical approval: No. 1143/19.04.2021.

## 3. Results

**Figure 1:**
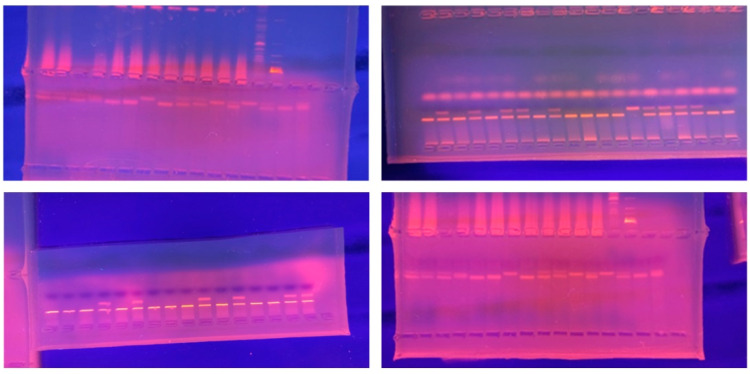
Part of the results on agarose gel samples.

In this study, we aim to assess the significance of genetic polymorphisms in *IL-10* at positions *−592 (rs1800872)* and *−1082 (rs1800896)*. All participants provided informed consent and met the specified inclusion criteria. Some agarose gel samples are represented at Figure 1.

### 3.1. Statistical Data for SNP of IL 10 −592 (rs 1800872) C>A

The descriptive statistics for SNP *IL 10* *−592* (*rs 1800872*) C>A is presented in Table 1.

The distribution of the genotype frequences of *IL-10* single-nucleotide polymorphism in *−592* position both in the healthy controls and the patient with periodontitis is shown in Table 2.

The statistical analysis revealed a predominance of heterozygosity and a minor frequency of the AA genotype exclusively within the periodontitis group. Notably, all four individuals with the AA genotype exhibited a high Bl/Age ratio (>1), a parameter related to the rapid progression of periodontitis. Table 3 presents the dominant genotype and allelic models for participants in both groups. Although the results for these four patients were not statistically significant due to the small sample size, it is worth noting that these individuals represent cases of severe periodontitis with a rapid progression rate (Stage III, Grade C). This observation leads us to hypothesize that the A-allele may be a potential risk factor for the development of periodontitis. 

The analysis of the genotype frequencies for the patients with periodontitis reveals that the observed data closely align with the expected values under the Hardy–Weinberg equilibrium model. The chi-squared test yielded a value of 0.2163, with no significant deviation from the equilibrium (*p* > 0.05). In the group of healthy controls, the chi-squared test yielded a value of 1.2, with two degrees of freedom, indicating no statistically significant deviation from the Hardy–Weinberg expectations (*p* > 0.05).

**Figure 2:**
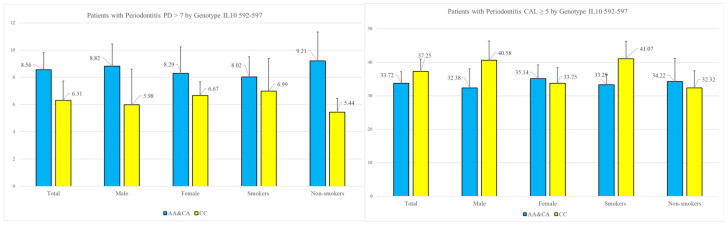
Key parameters of periodontitis—PD > 7 mm, and CAL ≥ 5 mm, grouped by genotype for −592 (*rs 1800872*), gender and smoking. PD—pocket depth; CAL—clinical attachment loss.

We evaluated the influence of the *IL-10* polymorphisms on the key parameters of periodontitis, including severe clinical attachment loss (CAL ≥ 5 mm) and the deepest periodontal pockets (PD > 7 mm), considering additional factors such as gender and tobacco smoking. The graphical distribution of these parameters is illustrated in Figure 2.

Regarding the deepest periodontal pockets, which often pose a significant clinical challenge, we found that the presence of at least one A-allele is associated with an increased risk of sites with probing depths (PDs) > 7 mm. Conversely, clinical attachment loss (CAL) ≥ 5 mm was more frequently observed in patients with the CC genotype, who also exhibited a greater number of sites with recession but shallower probing pocket depths.

### 3.2. Statistical Data for SNP of IL 10 −1082 (rs 1800896) A>G

Descriptive analysis for the SNP of *IL-10* at position *−1082 (rs1800896)* is presented in Table 4. Table 5 illustrates the distribution of the three genotypes among both groups—healthy individuals and patients with periodontitis.

For the SNP *IL 10* *−1082 (rs1800896) A>G*, we observed a differential distribution of the three genotypes. In the periodontitis group, the AA and AG genotypes were found in equal proportions, while the GG genotype was the least represented. Using the dominant model, we determined that individuals with at least one A-allele exhibit a heightened risk for periodontitis, with an odds ratio (OR) of 2.10 and a 95% confidence interval (CI) of 0.83 to 5.3 (Table 6).

**Figure 3:**
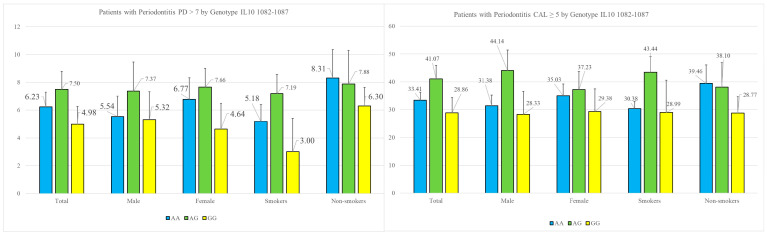
Key parameters of periodontitis—PD > 7 mm, and CAL ≥ 5 mm, grouped by genotype for *−1082* (*rs 1800896*), gender and smoking. PD—pocket depth; CAL—clinical attachment loss.

Regarding the periodontal parameters, namely probing depth (PD) > 7 mm and clinical attachment loss (CAL) ≥ 5 mm, we observed higher values in patients with at least one A-allele compared to those with the GG genotype. This trend was consistent regardless of gender and smoking status and is shown at Figure 3. Our analysis of genotype frequencies within the subgroup of patients with periodontitis revealed a close alignment with Hardy–Weinberg equilibrium expectations. The chi-squared test yielded a value of 0.544 with 2 degrees of freedom, which is not statistically significant (*p* > 0.05). Based on the chi-squared test, there is a statistically significant deviation from Hardy–Weinberg equilibrium in this population. The chi-squared value of 6.25, with two degrees of freedom and a *p*-value of approximately 0.043, indicates that the observed genotype frequencies differ significantly from those expected under equilibrium conditions.

The clinical findings and statistical data from our study suggest that the A-allele may be a significant factor in periodontitis, particularly its severe form, with respect to key clinical parameters such as probing depth (PD) > 7 mm and clinical attachment loss (CAL) ≥ 5 mm. The cumulative analysis of the A-allele frequency revealed a statistically significant association between the presence of at least one A-allele and the parameter PD > 7 mm, with a *p*-value of 0.047 (Table 7). These results support the hypothesis that the A-allele may serve as a risk factor for severe periodontitis.

## 4. Discussion

Periodontitis exemplifies a multifactorial disease resulting from chronic inflammation, driven by a complex of periodontopathogens and the disbalance in the host’s immune response. This imbalance can activate genes involved in immune, regenerative, and metabolic processes. These mechanisms are significant factors influencing the clinical manifestation of gene polymorphisms. The host immune response is a highly dynamic process, playing a crucial role in the chronic course of inflammation. Disease progression is linked to various immune factors, multiple competing pathogens, dysbiosis, and epigenetic factors, which collectively contribute to systemic disease. An inadequate immune response, due to an imbalance between pro- and anti-inflammatory factors, may exacerbate the cumulative effect of inflammatory/infectious processes, thereby explaining the progression of periodontal disease [36]. 

To gain a comprehensive understanding of the etiopathogenesis of periodontitis, an increasing number of studies are turning their attention to the human genome, examining its characteristics and variations across diverse geographical regions. Research into single-gene polymorphisms has been extensive, particularly in relation to complex diseases such as rheumatoid arthritis, asthma, multiple sclerosis, and periodontitis. These investigations suggest that certain gene polymorphisms may also be linked to an increased risk of malignant diseases. While the scientific literature documents polymorphisms in various molecules, the most thoroughly studied are those involving pro- and anti-inflammatory cytokines. These gene polymorphisms can exhibit diverse expression patterns across different populations. In Bulgaria, for example, *IL-10* polymorphisms have been extensively studied in relation to multiple sclerosis, rheumatoid arthritis, and lupus erythematosus, but to the best of the authors’ knowledge, not in relation to periodontitis. This research underscores the importance of genetic factors in the susceptibility to and progression of these diseases, offering insights into potential therapeutic targets and personalized treatment approaches [37,38,39]. 

We compared the findings of the current study with those in the existing literature across various populations to contextualize our results. Interleukin-10 (IL-10) is a key anti-inflammatory cytokine, and its gene polymorphisms are frequently studied, particularly at positions *−819 (rs1800871)*, *−1082 (rs1800896)*, and *−592 (rs1800872)*. These *IL-10* gene polymorphisms have been proposed as potential risk factors for periodontitis. However, evidence from previous genetic case–control studies has produced conflicting results regarding their association with the disease [40]. The *IL-10* gene is located on chromosome 1q31–q32 [41], and its polymorphisms have been implicated in a range of inflammatory, autoimmune, and malignant conditions. Associations have been reported with lymphoid leukemia [42], several cancers including lung cancer [43], ovarian cancer [44], colorectal cancer [45], and gastric cancer [45]. Additionally, these polymorphisms have been linked to tuberculosis [46], Behçet’s disease [47], diabetes mellitus [48], susceptibility to sepsis [49], and periodontitis [10]. This broad spectrum of associations highlights the complex role of IL-10 in immune regulation and disease susceptibility, underlining the need for further research to clarify its precise role in periodontitis.

Meta-analyses have suggested a significant association between the *rs1800872* polymorphism of the *IL-10* gene and periodontitis, observed in both the dominant model (CA + AA vs. CC) and the allelic model (A allele vs. C allele) across Caucasian, Asian, and mixed populations [21,50,51]. Similarly, for the *rs1800896* polymorphism, meta-analyses have reported associations with periodontitis in the dominant model (AA + AG vs. GG) and the allelic model (A allele vs. G allele). However, the findings based on ethnicity show variability: *rs1800896* was related to periodontitis in the Iranian population [41] but not in the Macedonian Caucasian population [26]. Conversely, a significant relationship between IL-10 and periodontitis was established in mixed populations [21]. Studies also indicate that IL-10 levels are often reduced in patients with severe periodontitis [28]. Furthermore, meta-analyses have highlighted an association between the *IL-10 −1082* single-nucleotide polymorphism (SNP), particularly the G-allele, and periodontitis in European and Latino populations [29]. In an Iranian cohort, Moudi et al. found that the G-allele of the *IL-10 −1082* SNP was linked to increased susceptibility to periodontitis. However, no statistically significant association was observed between the *IL-10 −592* SNP and periodontitis risk, despite a slightly higher prevalence of the C-allele in patients compared to healthy controls [41]. 

In a study on *IL-10* polymorphisms, Wang et al. highlighted the significance of the CC genotype in relation to periodontitis by linking the AA genotype and the A-allele to rapidly progressive periodontitis, a finding corroborated by our study [52]. Similarly, Toker et al. confirmed the association between the AA genotype and periodontal disease [53]. In an Italian sample, the A-allele was associated with an increased risk of developing periodontitis among patients and healthy controls [41]. The meta-analyses of 26 studies demonstrated a connection between the AA genotype and periodontitis in European populations, whereas the GG genotype was found to influence the progression of periodontitis in the Han population of China [40]. 

In a study on *IL-10* polymorphisms, Gamonal et al. emphasized the significance of the CC genotype in relation to periodontitis, highlighting a connection between the AA genotype and the A-allele with rapidly progressive periodontitis [52]. This finding is consistent with our study. Similarly, Toker et al. validated the association between the AA genotype and periodontal disease [53]. In an Italian cohort, the A-allele was linked to an increased risk of developing periodontitis among both patients and healthy controls [41]. A meta-analysis of 26 studies further supported the association between the AA genotype and periodontitis in European populations. In contrast, the GG genotype was found to influence the progression of periodontitis specifically in the Han population of China [40]. These findings underscore the complex role of *IL-10* polymorphisms in the susceptibility to and the progression of periodontitis across different populations and do not align with the data from our study.

Research on gene polymorphisms enables the identification of genetic factors that may predispose individuals to specific diseases, thereby facilitating the development of personalized diagnostic approaches. The variation in genetic factors across different populations underscores the importance of investigating gene polymorphisms in diverse molecular contexts. In the present study, we analyzed the prevalence of specific single-nucleotide polymorphisms (SNPs) in the *IL-10* gene at positions *−1082* and *−592*. Our findings revealed statistically significant associations between the A-allele and the severity of periodontitis, as measured by the periodontal parameter probing depth. Notably, the deepest periodontal pockets, which pose significant challenges in periodontitis treatment and are commonly linked to an increased risk of tooth loss, exhibited a strong correlation with the A-allele.

## 5. Conclusions

In this study, we examined the impact of *IL-10* single-nucleotide polymorphisms (SNPs) on the severity of periodontitis within a cohort of Bulgarian patients. Our findings revealed a significant association between specific *IL-10* SNPs alleles and the severity of periodontitis, underscoring the role of genetic factors in the pathogenesis of this condition. These results highlight the potential of *IL-10* SNPs as biomarkers for evaluating the risk and severity of periodontitis, which could facilitate the development of personalized treatment strategies and enable early intervention.

Our study emphasizes the importance of further research involving larger and more diverse populations to validate the relevance of *IL-10* SNPs as markers for periodontitis severity within the Bulgarian population and beyond. The identification of genetic markers associated with periodontitis not only advances our understanding of the disease mechanisms but also paves the way for targeted therapeutic approaches. Additionally, it is important to explore the interactions between *IL-10* SNPs and other genetic, environmental, and lifestyle factors to develop a comprehensive risk profile for periodontitis. Such advancements have the potential to lead to improved diagnostic tools, preventive measures, and ultimately, more effective management strategies that could reduce periodontitis-related morbidity.

## Figures and Tables

**Table 1:** General characteristics of all participants.

Variable		Share
Base (Number of Patients)	*N* *** = 102	
Gender	Male	46%
	Female	54%
Smoking habits	No	54%
	Yes	46%
SNP * of IL ** 10 *−592 (rs 1800872) C>A*	AA	4%
	CA	38%
	CC	58%
Periodontal status	Periodontitis	71%
	Periodontal health	29%

* SNP—single-nucleotide polymorphism. ** IL—interleukin. *** N—number.

**Table 2:** Bivariate distribution by gender, smoking habits, genetic polymorphism, and periodontal status for *IL 10 −592*.

Periodontal Status	Gender				Smoking Habits				IL ** 10/592 *(rs 1800872) C>A*					
	Male		Female		No		Yes		AA		CA		CC	
	*n*	%	*n*	%	*n*	%	*n*	%	*n*	%	*n*	%	*n*	%
Periodontitis	37	78.7%	35	63.6%	32	58.2%	40	85.1%	4	100.0%	29	74.4%	39	66.1%
Periodontal health	10	21.3%	20	36.4%	23	41.8%	7	14.9%	0	0.0%	10	25.6%	20	33.9%
Total	47	100.0%	55	100.0%	55	100.0%	47	100.0%	4	100.0%	39	100.0%	59	100.0%

** IL—interleukin.

**Table 3:** Distribution by genotypes and alleles.

	Periodontal Status								
Genotype	Periodontitis		Periodontal Health		*p* *		*p* **	OR	CI (90%)
AA + CA	33	45.83%	10	33.33%	0.24	(AA + CA) vs. CC	0.28	1.69	(0.8; 3.57)
CC	39	54.17%	20	66.67%					
**Allele**	**Periodontitis**		**Periodontal Health**		*p* *		*p* **	**OR**	**CI (90%)**
A	37	25.69%	10	16.67%	0.16	A vs. C	0.2	1.73	(0.9; 3.31)
C	107	74.31%	50	83.33%					

* χ^2^ test. ** Fisher exact test.

**Table 4:** General characteristics of all participants.

Variable		Share
Base (Number of Patients)	*N* *** = 89	
Gender	Male	45%
	Female	55%
Smoking habits	No	52%
	Yes	48%
SNP * of IL ** 10/*−1082 (rs 1800896) A>G*	AA	44%
	AG	37%
	GG	19%
Periodontal status	Periodontitis	72%
	Periodontal health	28%

* SNP—single-nucleotide polymorphism. ** IL—interleukin. *** N—number.

**Table 5:** Bivariate distribution by gender, smoking habits, genetic polymorphism, and periodontal status for *IL 10 −1082*.

Periodontal Status	Gender				Smoking Habits				IL 10/*−1082 (rs 1800896) (A>G)*					
	Male		Female		No		Yes		AA		AG		GG	
	*n*	%	*n*	%	*n*	%	*n*	%	*n*	%	*n*	%	*n*	%
Periodontitis	32	80.0%	32	65.3%	27	58.7%	37	86.0%	27	69.2%	27	81.8%	10	58.8%
Periodontal health	8	20.0%	17	34.7%	19	41.3%	6	14.0%	12	30.8%	6	18.2%	7	41.2%
Total	40	100.0%	49	100.0%	46	100.0%	43	100.0%	39	100.0%	33	100.0%	17	100.0%

IL—interleukin.

**Table 6:** Distribution by genotypes and alleles.

	Periodontal Status								
Genotype	Periodontitis		Periodontal Health		*p* *		*p* **	OR	CI (90%)
AA	27	42.19%	12	48.00%	0.20	AA vs. AG	0.28	0.50	(0.2; 1.28)
AG	27	42.19%	6	24.00%		AA vs. GG	0.54	1.58	(0.58; 4.24)
GG	10	15.63%	7	28.00%		AG vs. GG	0.09	3.15	(1.05; 9.46)
**Genotype**	**Periodontitis**		**Periodontal Health**		*p* *		*p* **	**OR**	**CI (90%)**
AA + AG	54	84.38%	18	72.00%	0.18	(AA + AG) vs. GG	0.23	2.10	(0.83; 5.3)
GG	10	15.63%	7	28.00%					
**Allele**	**Periodontitis**		**Periodontal Health**		*p* *		*p* **	**OR**	**CI (90%)**
A	81	63.28%	30	60.00%	0.68	A vs. G	0.73	1.15	(0.65;2.02)
G	47	36.72%	20	40.00%					

* χ^2^ test. ** Fisher exact test.

**Table 7:** Cumulative analysis by allele presence.

Clinical Parameter	IL 10/592 *(−597) (rs 1800872) C>A*				
	AA&CA		CC		
	N ***	Mean	N ***	Mean	*p*-Value
PD * > 7 (%)	33	8.561	39	6.313	0.246
CAL ** ≥ 5 (%)		33.715		37.252	0.490
**Clinical Parameter**	**IL 10/*−1082 (−1087) (rs 1800896) T>C (1082A>G)***				
	**AA**		**GG**		
	N ***	**Mean**	N ***	**Mean**	***p*-Value**
PD * > 7 (%)	27	6.226	10	4.980	0.523
CAL ** ≥ 5 (%)		33.407		28.857	0.436
**Clinical Parameter**	**AG**		**GG**		
	N ***	**Mean**	N ***	**Mean**	***p*-Value**
PD * > 7 (%)	27	7.496	10	4.980	0.275
CAL ** ≥ 5 (%)		41.066		28.857	0.174
**Clinical Parameter**	**Allele Combination**				
	**At Least One A-Allele**				
	N ***	**Mean**	N ***	**Mean**	***p*-Value**
PD * > 7 (%)	27	8.263	37	5.3295	**0.047**
CAL ** ≥ 5 (%)		35.789		36.028	0.963

* PD—pocket depth. ** CAL—clinical attachment loss. *** N—number.

## Data Availability

All available data are published in the manuscript.
